# Confirmed Hypoallergenicity of a Novel Whey-Based Extensively Hydrolyzed Infant Formula Containing Two Human Milk Oligosaccharides

**DOI:** 10.3390/nu11071447

**Published:** 2019-06-26

**Authors:** Anna Nowak-Wegrzyn, Laura Czerkies, Kemuel Reyes, Barbara Collins, Ralf G. Heine

**Affiliations:** 1 Icahn School of Medicine at Mount Sinai, New York, NY 10029-5674, USA; 2 Nestlé Nutrition, Arlington, VA 22209, USA; 3 Nestlé Health Science, CH-1800 Vevey, Switzerland; 4 Clinipace, Morrisville, NC 27560, USA

**Keywords:** cow’s milk protein allergy, food allergy, human milk oligosaccharides, infant formula, oral food challenge, whey

## Abstract

Background: We sought to determine whether an extensively hydrolyzed formula (EHF) supplemented with two human milk oligosaccharides (HMO) was tolerated by infants with cow’s milk protein allergy (CMPA). Methods: A whey-based EHF (Test formula) containing 2′fucosyl-lactose (2′FL) and lacto-N-neotetraose (LNnT) was assessed for clinical hypoallergenicity and safety. The Control formula was a currently marketed EHF without HMO. Children with CMPA, aged 2 months to 4 years, were assessed by double-blind, placebo-controlled food challenges (DBPCFC) to both formulas, in randomized order. If both DBPCFC were negative, subjects participated in a one-week, open food challenge (OFC) with the Test formula. Symptoms and adverse events were recorded. Hypoallergenicity was accepted if at least 90% (with 95% confidence intervals) of subjects tolerated the Test formula. Results: Of the 82 children with CMPA that were screened, 67 (intention-to-treat [ITT] cohort—mean age 24.5 ± 13.6 months; range 2–57; 45 [67.2%] male) were randomized to receive either the Test or the Control formula during the first DBPCFC. Of these, 64 children completed at least one DBPCFC (modified intention-to-treat [mITT] cohort). Three children were excluded due to protocol deviations (per protocol [PP] cohort; *n* = 61). There was one allergic reaction to the Test, and one to the Control formula. On the mITT analysis, 63 out of 64 (98.4%; 95% CI lower bound 92.8%), and on the PP analysis 60 out of 61 (98.4%; 95% CI lower bound 92.5%) participants tolerated the Test formula, confirming hypoallergenicity. Conclusion: The whey-based EHF supplemented with 2′FL and LNnT met the clinical hypoallergenicity criteria and can be recommended for the management of CMPA in infants and young children.

## 1. Introduction

Cow’s milk protein is a major food allergen in infants and young children [1,2]. Major milk allergens include caseins, as well as the whey proteins beta-lactoglobulin (BLG) and alpha-lactalbumin [2]. The prevalence of IgE-mediated cow’s milk protein allergy (CMPA) varies by region, with an overall incidence of challenge-proven CMPA of 0.54% of European children under 2 years of age. [3]. A recent US survey of parent-reported food allergy found a higher prevalence estimate of 4.3% for IgE-mediated CMPA in children under 2 years of age [4]. The prevalence of non-IgE-mediated CMPA is less well-defined [5,6]. Breast milk is the optimal source of nutrition for infants, but a hypoallergenic formula may be required in infants with CMPA who are not breastfed [7,8,9]. The American Academy of Pediatrics (AAP) considers a formula to be ‘hypoallergenic’ if at least 90% of infants with documented CMPA tolerated it under double-blind, placebo-controlled conditions [10]. These hypoallergenicity criteria are also endorsed by other allergy societies [8,9,11].

Human milk oligosaccharides (HMO) are a diverse group of non-digestible carbohydrates in breast milk, which exert a range of physiological functions and clinical benefits [12]. More than 150 HMO with different functionalities have been identified [13]. HMO provide the host-specific substrate for colonization of the infant’s gut with beneficial bifidobacteria (e.g., *Bifidobacterium infantis*) and lactobacilli, while suppressing potentially pathogenic phyla [14,15]. Until recently, HMO were not present in cow’s milk-based formula which may have negatively affected the early microbiome development and immune function of formula-fed infants [16,17]. Supplementation of standard infant formula with two HMO, 2′fucosyl-lactose (2′FL) and lacto-N-neotetraose (LNnT) has been shown to increase the number of bifidobacteria and production of short chain fatty acids in the gut [18]. This was associated with a significantly reduced rate of lower respiratory tract infections, compared to infants receiving non-supplemented infant formula [18]. In order to extend these HMO-related clinical benefits to infants with CMPA, an existing extensively hydrolyzed formula (EHF) was supplemented with 2′FL and LNnT. The aim of this study was to assess the tolerability and safety of this formula, and to document if this EHF with HMO meets the generally accepted hypoallergenicity criteria [10].

## 2. Materials and Methods

Infants and children between 2 months and 4 years of age with documented CMPA, who were otherwise healthy, were recruited at 12 study sites in the United States from July 2017–November 2018. The study protocol was approved by the Copernicus Group Independent Review Board (Cary NC, USA), and the Institutional Review Board of the Icahn School of Medicine at Mount Sinai, New York, USA. Written informed consent was obtained from the parents or legal guardians of the participating children. The study was prospectively registered (Clinicaltrials.gov identifier NCT03236207).

### 2.1. Inclusion and Exclusion Criteria

The diagnosis of CMPA was confirmed by one of the following inclusion criteria (within 6 months prior to enrollment)—(1) report of a convincing allergic reaction to cow’s milk or a milk-containing food product, in conjunction with presence of milk-specific serum IgE level > 0.7 kU_A_/L or a skin prick test to milk ≥5 mm [19]; (2) physician-supervised oral food challenge that elicited objective immediate allergic symptoms; (3) milk-specific serum IgE ≥ 15 kU_A_/L for children ≥1 year or ≥5 kU_A_/L, if younger than 1 year [20,21]; or (4) a skin prick test wheal to cow’s milk extract ≥10 mm [19,22]. All subjects had followed a strict cow’s milk protein-free elimination diet prior to enrolment. 

Exclusion criteria for participation in the study were any breastfeeding at the time of enrolment, treatment with an amino acid-based formula or current participation in another clinical study. Patients with any chronic medical diseases, major chromosomal or congenital anomalies, major gastrointestinal diseases or abnormalities (other than CMPA), immunodeficiency, unstable asthma, severe uncontrolled eczema or a previous severe anaphylactic reaction (requiring 2 or more doses of epinephrine) to cow’s milk, within the last two years, were also excluded. 

### 2.2. Test and Control Infant Formulas

The Test infant formula was a 100% whey-based EHF, supplemented with the HMO 2′FL (1.0 g/L) and LNnT (0.5 g/L). The 2′FL and LNnT supplements were free of residual milk proteins, as confirmed by gel electrophoresis (SDS-PAGE, Pharmacia PhastSystem™ with silver staining) [23] and high-sensitivity ELISA testing (Euroclone Spa, Pero, Italy; limits of quantification 0.01 mg/kg for BLG and 0.2 mg/kg for casein). The Control infant formula was a commercially available EHF without HMO (Althéra^®^, Nestlé Health Science, Vevey, Switzerland), which has been confirmed to be hypoallergenic [24]. Both whey hydrolysates were manufactured with a non-porcine enzyme blend. The protein/peptide content of the Test formula was 2.20 g/100 kcal, compared to 2.47 g/100 kcal for the Control formula. The macronutrient and micronutrient profiles of both formulas were otherwise almost identical. Both formulas contained highly purified lactose, which provided 52% of the total carbohydrates (29 g lactose in 100 g formula powder or 3.8 g in 100 mL prepared formula). Taste and appearance of the Test and Control formulas were indistinguishable.

### 2.3. Double-Blind, Placebo-Controlled Food Challenge Procedure

Subjects underwent double-blind, placebo-controlled food challenges (DBPCFC) with the Test and Control formulas in a blinded, cross-over fashion [24]. The Test and Control products were given in randomized order, with the first challenge session occurring within 3–28 days after enrolment, and the second challenge within 2–7 days of the first session. Subjects were provided with an emergency treatment plan and prescriptions for emergency medications, as required. Antihistamine use (except eye drops) during the 7 days prior to the food challenge, or oral steroid use within 14 days prior to enrolment were not permitted. Subjects were asked to fast for 1 h prior to each DBPCFC session. A light meal until 2 h prior to each session was permitted. For subjects ≤1 year of age, the initial dose was a lip smear with the assigned infant formula, followed by oral doses of 5 mL, 10 mL, 20 mL, 30 mL, 30 mL, 35 mL, and 50 mL at 10–15 min intervals (total volume 180 mL). For subjects >1 year of age, the initial dose was a lip smear, followed by 5 mL, 10 mL, 25 mL, 45 mL, 45 mL, 45 mL, and 65 mL orally at 10–15 min intervals (total volume 240 mL). A DBPCFC was considered evaluable if subjects had consumed a minimum of 100 mL of formula. A minimum observation period of 1 h after the final dose was required. Any allergic signs or symptoms (cutaneous, gastrointestinal, respiratory, or cardiovascular) attributable to the challenge formula were documented on a standardized DBPCFC data collection form. The challenge outcome was assessed according to pre-defined pass/fail criteria for each symptom during the DBPCFC, in line with the recommendations of the Adverse Reactions to Food Committee of the American Academy of Allergy, Asthma, and Immunology [25].

### 2.4. Open-Label, Test Formula Challenge at Home

If a subject successfully passed both sessions of the DBPCFC, a one-week open challenge with the Test formula was required to assess tolerance and confirm the absence of any delayed allergic reactions. Subjects were given the Test formula and instructed to drink a minimum of 240 mL daily, for a period of one week (7–9 days). Daily formula intake as well as the following clinical parameters were recorded—(1) daily stool frequency, color, consistency, and odor; (2) frequency of flatulence, (3) frequency of spitting-up and/or vomiting, (4) any potential allergic symptoms, and (5) any other adverse or serious adverse events.

### 2.5. Statistical Analysis Plan

The primary objective of this clinical trial was to determine whether the Test formula met the criteria for hypoallergenicity, aiming to demonstrate that at least 90% of infants or children with confirmed CMPA do not develop allergic reactions during a DBPCFC. The study design was based on the guidance provided by the AAP which states that, “To establish the risk of hypersensitivity in infants, carefully conducted preclinical studies must be performed that demonstrate a formula may be hypoallergenic. The formula needs to be tested in infants with hypersensitivity to cow’s milk or cow’s milk-based formula and the findings verified by properly conducted elimination-challenge tests. These tests should, at a minimum, ensure with 95% confidence that 90% of infants with documented cow’s milk allergy will not react with defined symptoms to the formula under double-blind, placebo-controlled conditions [10].”

Baseline clinical and demographic characteristics were summarized using descriptive statistics (the number of subjects, mean, standard deviation, median, minimum, and maximum). The number and percentage of subjects with no allergic reactions were summarized by formula (Test versus Control) and group allocation (‘Test → Control’; ‘Control → Test’). One-sided, exact lower bounds were provided for the point estimates of the proportion of subjects with no allergic reactions to formula. If the exact 95% confidence interval lower bound for the proportion of subjects without allergic reactions at the end of the study was greater than 90%, hypoallergenicity was confirmed [10].

*Definition of study populations*: Three analysis populations were defined. The intention-to-treat (ITT) population included all randomized subjects who took any amount of the study formula. The modified ITT population (mITT) was defined as all randomized subjects who completed at least one DBPCFC. A subject was included in the Per Protocol (PP) population if both the Test and the Control DBPCFC were completed. The determination of hypoallergenicity was based on the mITT and PP populations. For the 1-week open challenge with the Test formula, descriptive statistics were performed to summarize formula intake and clinical symptoms. All statistical analyses were performed using the SAS statistical software (Version 9.3). 

*Sample size calculation*: A minimum of 61 subjects in the PP cohort was the recruitment target as it allowed for 2 reactions in the Test group, while still meeting the AAP hypoallergenicity criteria. A planned interim analysis was scheduled after 35 completed subjects. If either no allergic reactions had occurred (meeting the AAP criteria), or if more than two reactions occurred in the Test group (failing AAP criteria), the study would be stopped. If it was known that there was 1 reaction to both formulas (one subject reacting to both formulas), the interim analysis would not be performed and the study would continue to 61 subjects. In case of no allergic reactions in the Test group of 35 subjects, the adjusted one-sided exact 95% lower bound confidence interval (CI) would be 90.1% [10]. The maximum adjusted sample size assuming a linear spending function with one interim analysis at information fraction *t* = 0.53 (35/66) was set at 66. In the case where the interim analysis was performed but the decision was made to not stop the study, it would continue to 66 subjects. In case of one or two allergic reactions in each group (and without performing an interim analysis), a sample size of 61 subjects would be sufficient to meet the AAP criteria (95% lower bound CI >90%).

## 3. Results

Of the 82 children with CMPA screened in 10 of the 12 study sites, 67 (intention-to-treat [ITT] cohort—mean age 24.5 ± 13.6 months; range 2–57; 45 [67.2%] male) were randomized to receive either the Test or Control formula during the first DBPCFC. 

**Figure 1:**
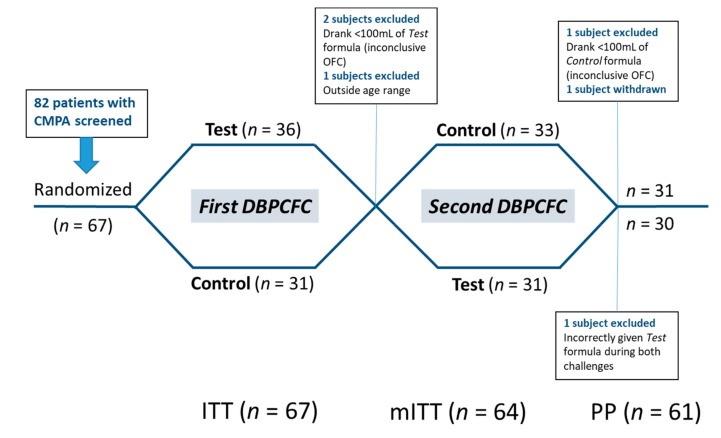
Study flow chart. Patients were allocated to perform two double-blind, placebo-controlled food challenges (DBPCFC) with the Test and Control formula in randomized order. ITT—Intention-to-treat; mITT—modified intention-to-treat; PP—per protocol analysis cohorts.

Thirty-six subjects were randomized to receive the Test formula first (‘Test → Control’), and 31 to receive the Control first (‘Control → Test’). In the ‘Test → Control’ group, 2 subjects were not able to consume enough infant formula (<100 mL) during the DBPCFC with the Test formula and one subject was outside the age range—these 3 subjects were excluded from the per protocol (PP) analysis. The analysis was performed with the remaining 64 subjects (modified intention-to-treat cohort; mITT). One patient erroneously completed both DBPCFC with the Test formula. This patient was included in the mITT analysis but excluded from the PP analysis due to a major protocol deviation [26]. Two subjects in the ‘Test → Control’ group withdrew before completing the second DBPCFC. The remaining 61 patients completed both DBPCFC and comprised the per protocol (PP) analysis cohort. The study flow and analysis cohorts are summarized in Figure 1. 

### 3.1. Modified Intention-To-Treat (mITT) Analysis

Sixty-four patients (mean age 24.1 ± 13.2 months) were included in the mITT analysis. The age distribution of subjects and the enrolment criteria are summarized in Table 1. Most subjects in the study identified as Caucasian/White (*n =* 36; 56.3%), followed by subjects of Black/African American (*n =* 29; 45.3%), Asian (*n =* 2; 3.1%), and native Hawaiian/Pacific Islander (*n =* 1; 1.6%) family origins. 

**Figure 2:**
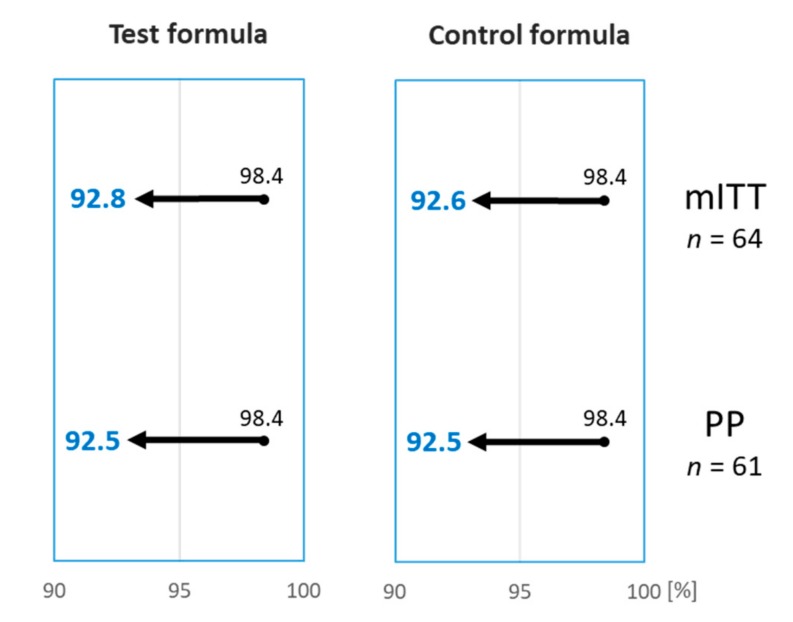
The percentage of subjects tolerating the Test and the Control formulas was 98.4%. Arrows indicate the 95% lower bound interval which was >90% for both Test and Control formula, on the modified intention-to-treat (mITT) and per-protocol (PP) analysis, confirming hypoallergenicity according to the criteria set out by the American Academy of Pediatrics [10].

A 12-month-old girl reacted during both DPBCFC, with widespread urticaria and an erythematous rash, but no other systemic clinical features, after ingesting a total of 165 mL of the Test and 85 mL of the Control formula during the first and second DBPCFC, respectively. The reactions settled after treatment with an antihistamine. Based on the DBPCFC outcomes of the mITT cohort, 63 out of 64 subjects (98.4%; 95% CI lower bound 92.8%) tolerated the Test formula, and 61 out of 62 subjects (98.4%; 95% CI lower bound 92.6%) tolerated the Control formula (Table 2). The Test formula, therefore, met the defined hypoallergenicity criteria [10]. The hypoallergenicity of the Control formula was also reconfirmed, although this was not a required study endpoint (Figure 2).

### 3.2. Per-Protocol (PP) Analysis

In total, 61 subjects completed both DBPCFC with the Test and Control formulas, as randomized, and were considered part of the PP population. 60 out of 61 (98.4%; 95% lower bound CI 92.5%) subjects tolerated the Test formula, and 60 out of 61 (98.4%; 95% lower bound CI 92.5%) subjects tolerated the Control formula. Based on these results, the hypoallergenicity criteria was confirmed for both the Test and the Control formula (Figure 2).

### 3.3. Open-Label, Test Formula Challenge at Home

Sixty-two patients completed two DBPCFC, of whom 61 completed the open-label home challenge phase with the Test formula. The patient who failed both DBPCFC did not proceed with the open challenge. Fifty-five (90.2%) subjects consumed a minimum of 240 mL of the Test formula/day over days 1 to 7. The average amount of the formula consumed during the open challenge was 302 ± 161mL/day. Two patients reported gastrointestinal symptoms. One subject vomited on Day 1 of the home challenge but completed the home challenge without further problems. Another patient developed diarrhea on the last day of the challenge, which the site investigator attributed to gastroenteritis. The episode resolved after 4 days. Otherwise, no significant gastrointestintal symptoms (flatulence, abnormal stool frequency/consistency, increased spitting-up or vomiting) were reported. There were no reactions that warranted early discontinuation of the open formula challenge. No serious adverse events occurred during the entire study.

## 4. Discussion

We present the first hypoallergenicity trial of an HMO-supplemented EHF intended for the management of infants and young children with CMPA. Levels of 2′FL and LNnT in the EHF were comparable to those found in human milk [27,28]. Hypoallergenicity was confirmed for the HMO-supplemented EHF, and also re-confirmed for the Control formula containing the same whey hydrolysate [10]. HMO are manufactured by biofermentation from lactose, which, in theory, might introduce a risk of residual milk allergen contamination. However, several purification steps are involved to remove potential residual proteins. Laboratory analysis of 2′FL and LNnT batches by high-sensitivity ELISA, as well as gel electrophoresis (SDS-PAGE), revealed no evidence of residual milk allergens. Importantly, the current hypoallergenicity trial found no increased rates of clinical reactions, compared to the non-HMO Control formula. These findings suggest that supplementation of EHF with 2′FL and LNnT is safe in children with CMPA.

HMO are a mixture of non-digestible carbohydrates that make up the third biggest component in breast milk, after lactose and lipids [29]. While the benefits of HMO have been known for several decades, the production of HMO that are identical to those found in breast milk has only recently become technically feasible. Two types of HMO have so far been added to the infant formula—a fucosylated HMO, 2′FL, and a neutral, non-fucosylated HMO, LNnT [13,30]. Concentrations of HMO in breast milk vary according to Lewis blood group and fucosyltransferase (FUT2 and FUT3) secretor status [27,28]. HMO form the preferred substrate for bifidobacteria and provide beneficial effects on the developing microbiome of breastfed infants [14,31]. In contrast to galacto-oligosaccharides (GOS), HMO suppress potential gut pathogens, such as *Enterobacteriaceae* and enteric viruses, thereby providing protection against enteric infection [16,32]. HMO are absent from cow’s milk, and HMO supplementation of milk-based infant formulas might reduce the risk of enteric infection. In addition, HMO have been shown to positively affect gut epithelial integrity, apoptosis, and intestinal permeability [33]. Of particular relevance for infants with CMPA are the immune modulating properties of HMO, including reduced antigen–antibody complex-induced chemokine release [34]. Preclinical data in a mouse model of CMPA suggest that HMO might attenuate allergic symptoms [35]. Furthermore, preclinical experiments showed that HMO might promote tolerance development via interaction with dendritic cells [36].

Besides HMO, both study formulas contained highly purified lactose, which is another microbiome-modifying substrate. Lactose is the main carbohydrate in breast milk [37]. In infancy, lactose is not completely absorbed and reaches the colon where it is fermented to short chain fatty acids, including butyrate. This makes lactose a conditional prebiotic in infants, with related beneficial effects on the early gut microbiome development [38]. Similar to the effects of HMO, lactose in EHF has been shown to increase counts of fecal bifidobacteria and lactobacilli [39]. Infants with CMPA have an underlying dysbiosis with reduced fecal microbial biodiversity, and we hypothesize that the presence of both lactose and HMO in the EHF has beneficial synergistic effects on the developing gut microbiome and immune system of infants with CMPA [40,41].

The present study design was based on the guidance for a clinical hypoallergenicity trial provided by the AAP [10]. This included assessment by DBPCFC to a Test formula and a Control formula with proven hypoallergenicity in patients with CMPA, in a randomized study with adequate statistical power. A limitation of the present study was the lack of an oral milk challenge in order to confirm the diagnosis of ongoing CMPA. A diagnostic DBPCFC at the study entry was omitted, in order to minimize the clinical burden on study subjects and their families. Instead, the inclusion criteria were based on a recent history of a convincing allergic reaction to cow’s milk, in conjunction with evidence of IgE-sensitization to cow’s milk, or high-level sensitization above the 95%-predictive diagnostic decision points for CMPA [19,21].

In conclusion, the hypoallergenicity of this novel EHF supplemented with two HMO was confirmed by DBPCFC in children with CMPA, in line with the established guidelines for hypoallergenic formulas [10]. This provided clinical evidence that the formula can be recommended for the management of infants and young children with CMPA. Further studies on the effects of HMO on long-term clinical outcomes are needed.

## Figures and Tables

**Table 1:** Age range and diagnostic criteria for cow’s milk protein allergy (CMPA) in the modified intention-to-treat (mITT) cohort (*n =* 64).

Mean age at enrollment (months)	24.1 ± 13.2
**Age distribution(*n* [%])** <12 months	8 (12.5%)
12–36 months	39 (60.9%)
>36 months	17 (26.6%)
**Diagnostic criteria for CMPA (n [%])**	
Reported convincing allergic symptoms following ingestion of cow’s milk or milk-containing food product and presence of milk-specific serum IgE (>0.7 kU_A_/L), or positive skin prick test (wheal > 5 mm)	58 (90.6%)
Milk-specific serum IgE level ≥ 15 kU_A_/L [20,21] or skin prick test wheal ≥ 10 mm (>95% positive predictive diagnostic decision points) [19]	6 (9.4%)

**Table 2:** Outcome of DBPCFC to the Test and Control formula by group allocation in the modified intention-to-treat (mITT) cohort. The 95% confidence interval lower bound was greater than 90% for both formulas.

	Challenge Outcome	DBPCFC 1 *n* [%]	DBPCFC 2 *n* [%]	Total *n* [%]	95% CI Lower Bound
Test Formula	Positive	1 (3.0%)	0 (0.0%)	1 (1.6%)	92.8%
	Negative	32 (97.0%)	31 (100%)	63 (98.4%)	
Control Formula	Positive	0 (0.0%)	1 (3.2%)	1 (1.6%)	92.6%
	Negative	31 (100%)	30 (96.8%)	61 (98.4%)	

## Abbreviations

2′FL	2′fucosyl-lactose
AAP	American Academy of Pediatrics
BLG	Beta-lactoglobulin
CI	Confidence interval
CMPA	Cow’s milk protein allergy
DBPCFC	Double-blind, placebo-controlled food challenge
EHF	Extensively hydrolyzed formula
GOS	Galacto-oligosaccharide(s)
HMO	Human milk oligosaccharide(s)
ITT	Intention-to-treat
mITT	Modified intention-to-treat
LNnT	Lacto-N-neotetraose
PP	Per protocol
SDS-PAGE	Sodium dodecyl sulphate-polyacrylamide gel electrophoresis
SPT	Skin prick testing

## References

[1] Sicherer S.H. (2011). Epidemiology of food allergy. J. Allergy Clin. Immunol..

[2] Heine R.G., Elsayed S., Hosking C.S., Hill D.J. (2002). Cow’s milk allergy in infancy. Curr. Opin. Allergy Clin. Immunol.

[3] Schoemaker A.A., Sprikkelman A.B., Grimshaw K.E., Roberts G., Grabenhenrich L., Rosenfeld L., Siegert S., Dubakiene R., Rudzeviciene O., Reche M. (2015). Incidence and natural history of challenge-proven cow’s milk allergy in European children—EuroPrevall birth cohort. Allergy.

[4] Gupta R.S., Warren C.M., Smith B.M., Blumenstock J.A., Jiang J., Davis M.M., Nadeau K.C. (2018). The Public Health Impact of Parent-Reported Childhood Food Allergies in the United States. Pediatrics.

[5] Koletzko S., Heine R.G. (2015). Non-IgE mediated cow’s milk allergy in EuroPrevall. Allergy.

[6] Venter C., Brown T., Meyer R., Walsh J., Shah N., Nowak-Wegrzyn A., Chen T.X., Fleischer D.M., Heine R.G., Levin M. (2017). Better recognition, diagnosis and management of non-IgE-mediated cow’s milk allergy in infancy: iMAP-an international interpretation of the MAP (Milk Allergy in Primary Care) guideline. Clin. Transl Allergy.

[7] Groetch M., Nowak-Wegrzyn A. (2013). Practical approach to nutrition and dietary intervention in pediatric food allergy. Pediatr. Allergy Immunol..

[8] Fiocchi A., Brozek J., Schünemann H., Bahna S.L., von Berg A., Beyer K., Bozzola M., Bradsher J., Compalati E., Ebisawa M. (2010). World Allergy Organization (WAO) Diagnosis and Rationale for Action against Cow’s Milk Allergy (DRACMA) Guidelines. World Allergy Organ. J..

[9] Muraro A., Werfel T., Hoffmann-Sommergruber K., Roberts G., Beyer K., Bindslev-Jensen C., Cardona V., Dubois A., duToit G., Eigenmann P. (2014). EAACI Food Allergy and Anaphylaxis Guidelines Group. EAACI food allergy and anaphylaxis guidelines: Diagnosis and management of food allergy. Allergy.

[10] American Academy of Pediatrics (2000). Committee on Nutrition. Hypoallergenic infant formulas. Pediatrics.

[11] Koletzko S., Niggemann B., Arato A., Dias J.A., Heuschkel R., Husby S., Mearin M.L., Papadopoulou A., Ruemmele F.M., Staiano A. (2012). European Society of Pediatric Gastroenterology, Hepatology and Nutrition. Diagnostic approach and management of cow’s-milk protein allergy in infants and children: ESPGHAN GI Committee practical guidelines. J. Pediatr. Gastroenterol. Nutr..

[12] Vandenplas Y., Berger B., Carnielli V.P., Ksiazyk J., Lagstrom H., Sanchez Luna M., Migacheva N., Mosselmans J.M., Picaud J.C., Possner M. (2018). Human Milk Oligosaccharides: 2’-Fucosyllactose (2’-FL) and Lacto-N-Neotetraose (LNnT) in Infant Formula. Nutrients.

[13] Bode L. (2015). The functional biology of human milk oligosaccharides. Early Hum. Dev..

[14] Marcobal A., Sonnenburg J.L. (2012). Human milk oligosaccharide consumption by intestinal microbiota. Clin. Microbiol. Infect..

[15] Ramani S., Stewart C.J., Laucirica D.R., Ajami N.J., Robertson B., Autran C.A., Shinge D., Rani S., Anandan S., Hu L. (2018). Human milk oligosaccharides, milk microbiome and infant gut microbiome modulate neonatal rotavirus infection. Nat. Commun.

[16] Gibson G.R., Wang X. (1994). Regulatory effects of bifidobacteria on the growth of other colonic bacteria. J. Appl. Bacteriol..

[17] Plaza-Diaz J., Fontana L., Gil A. (2018). Human Milk Oligosaccharides and Immune System Development. Nutrients.

[18] Puccio G., Alliet P., Cajozzo C., Janssens E., Corsello G., Sprenger N., Wernimont S., Egli D., Gosoniu L., Steenhout P. (2017). Effects of Infant Formula With Human Milk Oligosaccharides on Growth and Morbidity: A Randomized Multicenter Trial. J. Pediatr. Gastroenterol. Nutr..

[19] Sporik R., Hill D.J., Hosking C.S. (2000). Specificity of allergen skin testing in predicting positive open food challenges to milk, egg and peanut in children. Clin. Exp. Allergy.

[20] Sampson H.A. (2001). Utility of food-specific IgE concentrations in predicting symptomatic food allergy. J. Allergy Clin. Immunol..

[21] García-Ara C., Boyano-Martínez T., Díaz-Peña J.M., Martín-Munoz F., Reche-Frutos M., Martín-Esteban M. (2001). Specific IgE levels in the diagnosis of immediate hypersensitivity to cows’ milk protein in the infant. J. Allergy Clin. Immunol..

[22] Verstege A., Mehl A., Rolinck-Werninghaus C., Staden U., Nocon M., Beyer K., Niggemann B. (2005). The predictive value of the skin prick test weal size for the outcome of oral food challenges. Clin. Exp. Allergy.

[23] Heukeshoven J., Dernick R. (1988). Improved silver staining procedure for fast staining in PhastSystem Development Unit. I. Staining of sodium dodecyl sulfate gels. Electrophoresis.

[24] Nowak-Wegrzyn A., Czerkies L., Kuslys M., Nutten S., Simons P.J., Collins B., Heine R.G. (2019). Hypoallergenicity of a whey-based, extensively hydrolyzed infant formula prepared with nonporcine enzymes. Allergy.

[25] Sampson H.A., Gerth van Wijk R., Bindslev-Jensen C., Sicherer S., Teuber S.S., Burks A.W., Dubois A.E., Beyer K., Eigenmann P.A., Spergel J.M. (2012). Standardizing double-blind, placebo-controlled oral food challenges: American Academy of Allergy, Asthma & Immunology-European Academy of Allergy and Clinical Immunology PRACTALL consensus report. J. Allergy Clin. Immunol..

[26] Yelland L.N., Sullivan T.R., Voysey M., Lee K.J., Cook J.A., Forbes A.B. (2015). Applying the intention-to-treat principle in practice: Guidance on handling randomisation errors. Clin. Trials.

[27] Urashima T., Taufik E., Fukuda K., Asakuma S. (2013). Recent advances in studies on milk oligosaccharides of cows and other domestic farm animals. Biosci. Biotechnol. Biochem..

[28] Xu G., Davis J.C., Goonatilleke E., Smilowitz J.T., German J.B., Lebrilla C.B. (2017). Absolute Quantitation of Human Milk Oligosaccharides Reveals Phenotypic Variations during Lactation. J. Nutr..

[29] Ballard O., Morrow A.L. (2013). Human milk composition: Nutrients and bioactive factors. Pediatr. Clin. N. Am..

[30] Petschacher B., Nidetzky B. (2016). Biotechnological production of fucosylated human milk oligosaccharides: Prokaryotic fucosyltransferases and their use in biocatalytic cascades or whole cell conversion systems. J. Biotechnol..

[31] Matsuki T., Yahagi K., Mori H., Matsumoto H., Hara T., Tajima S., Ogawa E., Kodama H., Yamamoto K., Yamada T. (2016). A key genetic factor for fucosyllactose utilization affects infant gut microbiota development. Nat. Commun..

[32] Hoeflinger J.L., Davis S.R., Chow J., Miller M.J. (2015). In vitro impact of human milk oligosaccharides on Enterobacteriaceae growth. J. Agric. Food Chem..

[33] Holscher H.D., Bode L., Tappenden K.A. (2017). Human Milk Oligosaccharides Influence Intestinal Epithelial Cell Maturation In Vitro. J. Pediatr. Gastroenterol. Nutr..

[34] Zehra S., Khambati I., Vierhout M., Mian M.F., Buck R., Forsythe P. (2018). Human Milk Oligosaccharides Attenuate Antigen-Antibody Complex Induced Chemokine Release from Human Intestinal Epithelial Cell Lines. J. Food Sci..

[35] Castillo-Courtade L., Han S., Lee S., Mian F.M., Buck R., Forsythe P. (2015). Attenuation of food allergy symptoms following treatment with human milk oligosaccharides in a mouse model. Allergy.

[36] Xiao L., van De Worp W.R., Stassen R., van Maastrigt C., Kettelarij N., Stahl B., Blijenberg B., Overbeek S.A., Folkerts G., Garssen J. (2019). Human milk oligosaccharides promote immune tolerance via direct interactions with human dendritic cells. Eur. J. Immunol..

[37] Heine R.G., AlRefaee F., Bachina P., De Leon J.C., Geng L., Gong S., Madrazo J.A., Ngamphaiboon J., Ong C., Rogacion J.M. (2017). Lactose intolerance and gastrointestinal cow’s milk allergy in infants and children -common misconceptions revisited. World Allergy Organ. J..

[38] Szilagyi A. (2004). Redefining lactose as a conditional prebiotic. Can. J. Gastroenterol..

[39] Francavilla R., Calasso M., Calace L., Siragusa S., Ndagijimana M., Vernocchi P., Brunetti L., Mancino G., Tedeschi G., Guerzoni E. (2012). Effect of lactose on gut microbiota and metabolome of infants with cow’s milk allergy. Pediatr. Allergy Immunol..

[40] Thompson-Chagoyan O.C., Fallani M., Maldonado J., Vieites J.M., Khanna S., Edwards C., Dore J., Gil A. (2011). Faecal microbiota and short-chain fatty acid levels in faeces from infants with cow’s milk protein allergy. Int. Arch. Allergy Immunol..

[41] Berni Canani R., De Filippis F., Nocerino R., Paparo L., Di Scala C., Cosenza L., Della Gatta G., Calignano A., De Caro C., Laiola M. (2018). Gut microbiota composition and butyrate production in children affected by non-IgE-mediated cow’s milk allergy. Sci. Rep..

